# (4*R*,6*S*,7*S*,8*S*,8a*S*)-6-Ethyl-7,8-dihy­droxy-4-methyl-1,2,3,5,6,7,8,8a-octa­hydro­indolizin-4-ium iodide

**DOI:** 10.1107/S1600536811051099

**Published:** 2011-11-30

**Authors:** Viktor Vrábel, Július Sivý, Ľubomír Švorc, Peter Šafář, Jozefína Žužiová

**Affiliations:** a Institute of Analytical Chemistry, Faculty of Chemical and Food Technology, Slovak University of Technology, Radlinského 9, SK-812 37 Bratislava, Slovak Republic 81237; b Institute of Mathematics and Physics, Faculty of Mechanical Engineering, Slovak Technical University, Namestie slobody 17, SK-812 31 Bratislava, Slovak Republic 81231; c Institute of Organic Chemistry, Catalysis and Petrochemistry, Faculty of Chemical and Food Technology, Slovak University of Technology, Radlinského 9, SK-812 37 Bratislava, Slovak Republic 81237

## Abstract

The title compound, C_11_H_22_NO_2_^+^·I^−^, is a chiral mol­ecule with five stereogenic centres. The absolute configuration was assigned from the synthesis and confirmed by the structure determination. The central six-membered ring of the indolizine system adopts a chair conformation, with two atoms displaced by −0.690 (2) and 0.550 (2) Å from the plane of the other four atoms. The conformation of the pyrrolidine ring is close to that of an envelope, with the flap atom displaced by 0.563 (2) Å from the plane of the remaining four atoms. In the crystal, there are two O—H⋯I hydrogen bonds.

## Related literature

For the biological activity of indolizine derivatives, see: Gubin *et al.* (1992 ▶); Gupta *et al.* (2003 ▶); Malonne *et al.* (1998 ▶); Medda *et al.* (2003 ▶); Nardelli (1983 ▶); Pearson & Guo (2001 ▶); Ruprecht *et al.* (1989 ▶). For puckering analysis, see: Cremer & Pople (1975 ▶). For the preparation, see: Šafář *et al.* (2010 ▶). For related structures, see: Clark & Reid (1995 ▶); Pedersen (1967 ▶).
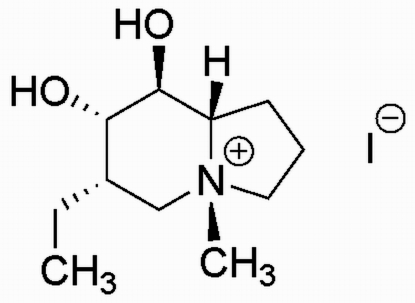


## Experimental

### 

#### Crystal data

C_11_H_22_NO_2_^+^·I^−^
*M* *_r_* = 327.20
Monoclinic, 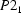

*a* = 8.18603 (14) Å
*b* = 10.82977 (14) Å
*c* = 8.19874 (13) Å
β = 110.3688 (19)°
*V* = 681.39 (2) Å^3^
*Z* = 2
Mo *K*α radiation
μ = 2.34 mm^−1^
*T* = 298 K
0.30 × 0.25 × 0.20 mm

#### Data collection

Oxford Diffraction Gemini R CCD diffractometer
Absorption correction: multi-scan (*CrysAlis PRO*; Oxford Diffraction, 2009 ▶) *T*_min_ = 0.520, *T*_max_ = 0.638
18648 measured reflections
3330 independent reflections
3176 reflections with *I* > 2σ(*I*)
*R*_int_ = 0.020

#### Refinement

*R*[*F* ^2^ > 2σ(*F* ^2^)] = 0.019
*wR*(*F* ^2^) = 0.049
*S* = 0.91
3330 reflections
141 parameters
1 restraint
H-atom parameters constrained
Δρ_max_ = 0.64 e Å^−3^
Δρ_min_ = −0.69 e Å^−3^
Absolute structure: Flack (1983 ▶), 1359 Friedel pairs
Flack parameter: −0.026 (17)

### 

Data collection: *CrysAlis PRO* (Oxford Diffraction, 2009 ▶); cell refinement: *CrysAlis PRO*; data reduction: *CrysAlis PRO*; program(s) used to solve structure: *SHELXS97* (Sheldrick, 2008 ▶); program(s) used to refine structure: *SHELXL97* (Sheldrick, 2008 ▶); molecular graphics: *DIAMOND* (Brandenburg, 2001 ▶); software used to prepare material for publication: *SHELXL97*, *PLATON* (Spek, 2009 ▶) and *WinGX* (Farrugia, 1999 ▶).

## Supplementary Material

Crystal structure: contains datablock(s) I, global. DOI: 10.1107/S1600536811051099/bq2323sup1.cif

Structure factors: contains datablock(s) I. DOI: 10.1107/S1600536811051099/bq2323Isup2.hkl

Supplementary material file. DOI: 10.1107/S1600536811051099/bq2323Isup3.cml

Additional supplementary materials:  crystallographic information; 3D view; checkCIF report

## Figures and Tables

**Table 1:** Hydrogen-bond geometry (Å, °)

*D*—H⋯*A*	*D*—H	H⋯*A*	*D*⋯*A*	*D*—H⋯*A*
O1—H1⋯I1^i^	0.82	2.80	3.6187 (18)	173
O2—H2⋯I1^ii^	0.82	2.67	3.4798 (16)	172

Symmetry codes: (i) 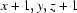; (ii) 
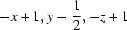
.

**Fig. 1.**
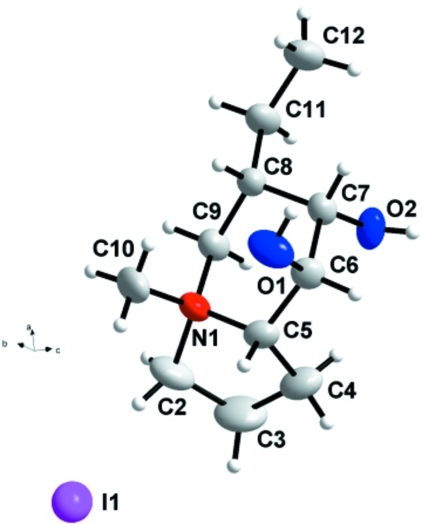
Molecular structure of the title compound showing the atom labeling scheme. Displacement ellipsoids are drawn at the 50% probability level (Brandenburg, 2001).

**Fig. 2.**
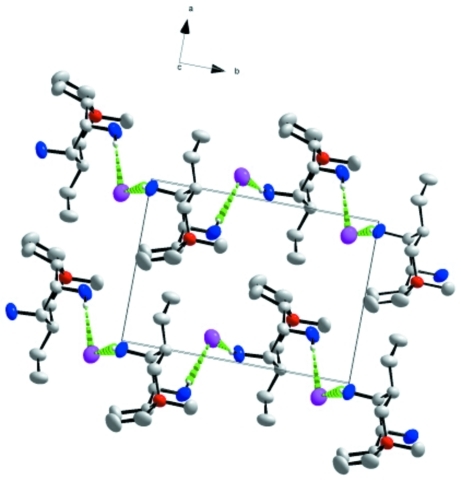
Packing view of the title compound. Molecular chains along b are generated by O–H···I hydrogen bonds which are shown as dashed lines. H atoms not involved in hydrogen bonding have been omitted.

## supplementary crystallographic information

### Comment

Indolizine derivatives have been found to possess a variety of biological activities such as antiinflammatory (Malonne *et al.*, 1998), antiviral (Medda *et al.*, 2003), and antitumor (Pearson & Guo, 2001) activities. They have also shown to be calcium entry blockers (Gupta *et al.*, 2003). As such, indolizines are important synthetic targets in view of developing new pharmaceuticals for the treatment of cardiovascular diseases (Gubin *et al.*, 1992) and HIV infections (Ruprecht *et al.*, 1989). Based on these facts and in continuation of our interest in developing simple and efficient route for the synthesis of novel indolizine derivatives, we report here the synthesis, molecular and crystal structure of the title compound. The molecular structure of the compound and the atom labeling scheme are shown in Fig. 1. The absolute configuration was established by synthesis and confirmed by the structure determination. The expected stereochemistry of atoms N1, C5, C6, C7 and C8 was confirmed as *R*,*S*,*S*,*S* and S, respectively (Fig.1). The central six-membered N-heterocyclic ring is not planar and adopts a chair conformation (Cremer & Pople, 1975). A calculation of least-squares planes shows that this ring is puckered in such a manner that the four atoms C6, C7, N1 and C9 are coplanar within 0.022 (2) Å, while atoms C8 and C5 are displaced from this plane on opposite sides, with out-of-plane displacements of -0.690 (2) and 0.550 (2) Å, respectively. The pyrrolidine ring attached to the indolizine ring system has envelope conformation, with atom N1 on the flap. The maximum deviation from planarity for N1 is -0.563 (2) Å. The two aromatic rings are almost perpendicular to each other. The dihedral angle between the plane of the four atoms C2, C3, C4 and C5 of pyrrolidine ring and the plane of the four atoms C6, C7, N1 and C9 forming the base of the chair conformation is 89.6 (1)°. Intermolecular O1–H1···I1 and O2–H2···I1 hydrogen bonds link the molecules into extended chains running along the *b* axis (Table 1. and Figure 2.).

### Experimental

The title compound was prepared according to a standard protocol described in literature (Šafář *et al.*, 2010).

### Refinement

All H atoms were positioned with idealized geometry using a riding model with C—H distances in the range 0.93 - 0.98 Å and O—H distance 0.82 Å. The *U*_iso_(H) values were set at 1.5*U*_eq_(*C*-methyl,O) and 1.2*U*_eq_(other C atoms)

### Crystal data

**Table:**

C_11_H_22_NO_2_^+^·I^−^	*F*(000) = 328
*M**_r_* = 327.20	*D*_x_ = 1.595 Mg m^−^^3^
Monoclinic, *P*2_1_	Mo *K*α radiation, λ = 0.71073 Å
Hall symbol: P 2yb	Cell parameters from 3330 reflections
*a* = 8.18603 (14) Å	θ = 3.6–29.4°
*b* = 10.82977 (14) Å	µ = 2.34 mm^−^^1^
*c* = 8.19874 (13) Å	*T* = 298 K
β = 110.3688 (19)°	Prism, colourless
*V* = 681.39 (2) Å^3^	0.30 × 0.25 × 0.20 mm
*Z* = 2	

### Data collection

**Table:**

Oxford Diffraction Gemini R CCD diffractometer	3330 independent reflections
Radiation source: fine-focus sealed tube	3176 reflections with *I* > 2σ(*I*)
graphite	*R*_int_ = 0.020
Detector resolution: 10.434 pixels mm^-1^	θ_max_ = 29.4°, θ_min_ = 3.6°
ω and φ scans	*h* = −11→11
Absorption correction: multi-scan (*CrysAlis PRO*; Oxford Diffraction, 2009)	*k* = −14→14
*T*_min_ = 0.520, *T*_max_ = 0.638	*l* = −11→10
18648 measured reflections	

### Refinement

**Table:**

Refinement on *F*^2^	Hydrogen site location: inferred from neighbouring sites
Least-squares matrix: full	H-atom parameters constrained
*R*[*F*^2^ > 2σ(*F*^2^)] = 0.019	*w* = 1/[σ^2^(*F*_o_^2^) + (0.0335*P*)^2^ + 0.2787*P*] where *P* = (*F*_o_^2^ + 2*F*_c_^2^)/3
*wR*(*F*^2^) = 0.049	(Δ/σ)_max_ < 0.001
*S* = 0.91	Δρ_max_ = 0.64 e Å^−^^3^
3330 reflections	Δρ_min_ = −0.69 e Å^−^^3^
141 parameters	Extinction correction: *SHELXL97* (Sheldrick, 2008), Fc^*^=kFc[1+0.001xFc^2^λ^3^/sin(2θ)]^-1/4^
1 restraint	Extinction coefficient: 0.044 (2)
Primary atom site location: structure-invariant direct methods	Absolute structure: Flack (1983), 1359 Friedel pairs
Secondary atom site location: difference Fourier map	Flack parameter: −0.026 (17)

### Special details

**Table:**

Geometry. All e.s.d.'s (except the e.s.d. in the dihedral angle between two l.s. planes) are estimated using the full covariance matrix. The cell e.s.d.'s are taken into account individually in the estimation of e.s.d.'s in distances, angles and torsion angles; correlations between e.s.d.'s in cell parameters are only used when they are defined by crystal symmetry. An approximate (isotropic) treatment of cell e.s.d.'s is used for estimating e.s.d.'s involving l.s. planes.
Refinement. Refinement of *F*^2^ against ALL reflections. The weighted *R*-factor *wR* and goodness of fit *S* are based on *F*^2^, conventional *R*-factors *R* are based on *F*, with *F* set to zero for negative *F*^2^. The threshold expression of *F*^2^ > σ(*F*^2^) is used only for calculating *R*-factors(gt) *etc*. and is not relevant to the choice of reflections for refinement. *R*-factors based on *F*^2^ are statistically about twice as large as those based on *F*, and *R*- factors based on ALL data will be even larger.

### Fractional atomic coordinates and isotropic or equivalent isotropic displacement parameters (Å^2^)

**Table:**

	*x*	*y*	*z*	*U*_iso_*/*U*_eq_	
C2	0.5506 (4)	0.1519 (3)	0.1098 (4)	0.0581 (7)	
H2A	0.4507	0.2056	0.0599	0.070*	
H2B	0.6055	0.1385	0.0237	0.070*	
C3	0.4953 (5)	0.0317 (3)	0.1635 (5)	0.0735 (9)	
H3A	0.5487	−0.0365	0.1238	0.088*	
H3B	0.3697	0.0229	0.1134	0.088*	
C4	0.5545 (4)	0.0321 (3)	0.3627 (4)	0.0538 (6)	
H4A	0.4590	0.0099	0.4012	0.065*	
H4B	0.6495	−0.0256	0.4122	0.065*	
C5	0.6143 (3)	0.1645 (2)	0.4157 (3)	0.0376 (4)	
H5	0.5115	0.2133	0.4092	0.045*	
C6	0.7478 (3)	0.1787 (2)	0.5989 (3)	0.0358 (4)	
H6	0.7088	0.1294	0.6787	0.043*	
C7	0.9331 (3)	0.13766 (19)	0.6181 (3)	0.0336 (4)	
H7	1.0133	0.1641	0.7321	0.040*	
C8	0.9902 (3)	0.19544 (19)	0.4777 (3)	0.0340 (4)	
H8	0.9914	0.2854	0.4913	0.041*	
C9	0.8601 (3)	0.1627 (2)	0.2992 (3)	0.0395 (5)	
H9A	0.8994	0.1978	0.2103	0.047*	
H9B	0.8562	0.0737	0.2853	0.047*	
C10	0.6736 (4)	0.3470 (2)	0.2554 (4)	0.0565 (7)	
H10A	0.7083	0.3703	0.1592	0.085*	
H10B	0.7517	0.3834	0.3603	0.085*	
H10C	0.5572	0.3757	0.2352	0.085*	
C11	1.1720 (3)	0.1536 (3)	0.4865 (3)	0.0477 (6)	
H11A	1.1688	0.0662	0.4598	0.057*	
H11B	1.2044	0.1977	0.3994	0.057*	
C12	1.3095 (3)	0.1765 (3)	0.6658 (4)	0.0586 (7)	
H12A	1.4233	0.1615	0.6611	0.088*	
H12B	1.2894	0.1220	0.7492	0.088*	
H12C	1.3020	0.2606	0.6998	0.088*	
N1	0.6791 (2)	0.20968 (18)	0.2730 (2)	0.0373 (4)	
O1	0.7466 (2)	0.30534 (17)	0.6448 (3)	0.0537 (5)	
H1	0.8265	0.3185	0.7367	0.080*	
O2	0.9442 (2)	0.00727 (14)	0.6053 (2)	0.0444 (4)	
H2	0.9405	−0.0249	0.6945	0.067*	
I1	0.11548 (2)	0.38255 (2)	0.028309 (17)	0.05615 (7)	

### Atomic displacement parameters (Å^2^)

**Table:**

	*U*^11^	*U*^22^	*U*^33^	*U*^12^	*U*^13^	*U*^23^
C2	0.0441 (13)	0.079 (2)	0.0373 (12)	−0.0013 (13)	−0.0032 (10)	−0.0131 (12)
C3	0.0651 (18)	0.070 (2)	0.072 (2)	−0.0226 (16)	0.0065 (16)	−0.0302 (17)
C4	0.0494 (13)	0.0441 (13)	0.0663 (17)	−0.0150 (11)	0.0180 (12)	−0.0109 (12)
C5	0.0291 (9)	0.0385 (10)	0.0444 (12)	−0.0017 (8)	0.0118 (8)	−0.0050 (9)
C6	0.0329 (9)	0.0395 (11)	0.0371 (10)	0.0043 (8)	0.0150 (8)	−0.0039 (9)
C7	0.0351 (10)	0.0352 (10)	0.0304 (10)	0.0048 (8)	0.0112 (8)	0.0018 (8)
C8	0.0288 (9)	0.0361 (10)	0.0351 (10)	0.0007 (8)	0.0088 (8)	0.0050 (8)
C9	0.0354 (10)	0.0501 (12)	0.0321 (10)	0.0021 (9)	0.0107 (8)	0.0033 (9)
C10	0.0488 (13)	0.0441 (14)	0.0606 (15)	0.0033 (9)	−0.0011 (11)	0.0162 (10)
C11	0.0309 (10)	0.0685 (17)	0.0435 (13)	0.0013 (10)	0.0127 (10)	0.0097 (11)
C12	0.0313 (11)	0.082 (2)	0.0570 (16)	−0.0028 (12)	0.0086 (11)	0.0059 (15)
N1	0.0317 (8)	0.0411 (9)	0.0318 (9)	−0.0007 (7)	0.0017 (7)	0.0010 (7)
O1	0.0487 (10)	0.0491 (10)	0.0534 (10)	0.0136 (8)	0.0054 (8)	−0.0195 (8)
O2	0.0575 (10)	0.0344 (8)	0.0492 (10)	0.0114 (7)	0.0286 (8)	0.0114 (7)
I1	0.06754 (11)	0.06164 (10)	0.04070 (9)	−0.00802 (11)	0.02063 (6)	−0.01043 (10)

### Geometric parameters (Å, °)

**Table:**

C2—C3	1.494 (5)	C8—C9	1.522 (3)
C2—N1	1.520 (3)	C8—C11	1.533 (3)
C2—H2A	0.9700	C8—H8	0.9800
C2—H2B	0.9700	C9—N1	1.510 (3)
C3—C4	1.533 (5)	C9—H9A	0.9700
C3—H3A	0.9700	C9—H9B	0.9700
C3—H3B	0.9700	C10—N1	1.493 (3)
C4—C5	1.528 (3)	C10—H10A	0.9600
C4—H4A	0.9700	C10—H10B	0.9600
C4—H4B	0.9700	C10—H10C	0.9600
C5—N1	1.523 (3)	C11—C12	1.529 (4)
C5—C6	1.527 (3)	C11—H11A	0.9700
C5—H5	0.9800	C11—H11B	0.9700
C6—O1	1.423 (3)	C12—H12A	0.9600
C6—C7	1.535 (3)	C12—H12B	0.9600
C6—H6	0.9800	C12—H12C	0.9600
C7—O2	1.421 (3)	O1—H1	0.8200
C7—C8	1.519 (3)	O2—H2	0.8200
C7—H7	0.9800		
			
C3—C2—N1	106.7 (2)	C7—C8—C11	113.04 (18)
C3—C2—H2A	110.4	C9—C8—C11	108.61 (19)
N1—C2—H2A	110.4	C7—C8—H8	108.5
C3—C2—H2B	110.4	C9—C8—H8	108.5
N1—C2—H2B	110.4	C11—C8—H8	108.5
H2A—C2—H2B	108.6	N1—C9—C8	112.42 (18)
C2—C3—C4	107.2 (2)	N1—C9—H9A	109.1
C2—C3—H3A	110.3	C8—C9—H9A	109.1
C4—C3—H3A	110.3	N1—C9—H9B	109.1
C2—C3—H3B	110.3	C8—C9—H9B	109.1
C4—C3—H3B	110.3	H9A—C9—H9B	107.9
H3A—C3—H3B	108.5	N1—C10—H10A	109.5
C5—C4—C3	104.9 (2)	N1—C10—H10B	109.5
C5—C4—H4A	110.8	H10A—C10—H10B	109.5
C3—C4—H4A	110.8	N1—C10—H10C	109.5
C5—C4—H4B	110.8	H10A—C10—H10C	109.5
C3—C4—H4B	110.8	H10B—C10—H10C	109.5
H4A—C4—H4B	108.8	C12—C11—C8	112.0 (2)
N1—C5—C6	113.69 (17)	C12—C11—H11A	109.2
N1—C5—C4	104.21 (19)	C8—C11—H11A	109.2
C6—C5—C4	115.0 (2)	C12—C11—H11B	109.2
N1—C5—H5	107.9	C8—C11—H11B	109.2
C6—C5—H5	107.9	H11A—C11—H11B	107.9
C4—C5—H5	107.9	C11—C12—H12A	109.5
O1—C6—C5	106.78 (18)	C11—C12—H12B	109.5
O1—C6—C7	110.46 (18)	H12A—C12—H12B	109.5
C5—C6—C7	114.51 (17)	C11—C12—H12C	109.5
O1—C6—H6	108.3	H12A—C12—H12C	109.5
C5—C6—H6	108.3	H12B—C12—H12C	109.5
C7—C6—H6	108.3	C10—N1—C9	110.1 (2)
O2—C7—C8	108.01 (17)	C10—N1—C2	109.6 (2)
O2—C7—C6	111.47 (18)	C9—N1—C2	109.31 (19)
C8—C7—C6	110.88 (17)	C10—N1—C5	112.8 (2)
O2—C7—H7	108.8	C9—N1—C5	111.69 (16)
C8—C7—H7	108.8	C2—N1—C5	103.01 (19)
C6—C7—H7	108.8	C6—O1—H1	109.5
C7—C8—C9	109.65 (17)	C7—O2—H2	109.5
			
N1—C2—C3—C4	13.6 (3)	C7—C8—C9—N1	60.6 (2)
C2—C3—C4—C5	10.0 (3)	C11—C8—C9—N1	−175.43 (19)
C3—C4—C5—N1	−29.6 (3)	C7—C8—C11—C12	−54.9 (3)
C3—C4—C5—C6	−154.8 (2)	C9—C8—C11—C12	−176.8 (2)
N1—C5—C6—O1	77.7 (2)	C8—C9—N1—C10	71.0 (2)
C4—C5—C6—O1	−162.2 (2)	C8—C9—N1—C2	−168.5 (2)
N1—C5—C6—C7	−44.9 (3)	C8—C9—N1—C5	−55.1 (2)
C4—C5—C6—C7	75.2 (3)	C3—C2—N1—C10	−152.2 (3)
O1—C6—C7—O2	168.90 (18)	C3—C2—N1—C9	87.0 (3)
C5—C6—C7—O2	−70.5 (2)	C3—C2—N1—C5	−31.8 (3)
O1—C6—C7—C8	−70.7 (2)	C6—C5—N1—C10	−78.1 (2)
C5—C6—C7—C8	49.9 (2)	C4—C5—N1—C10	155.86 (19)
O2—C7—C8—C9	66.0 (2)	C6—C5—N1—C9	46.5 (2)
C6—C7—C8—C9	−56.5 (2)	C4—C5—N1—C9	−79.5 (2)
O2—C7—C8—C11	−55.4 (2)	C6—C5—N1—C2	163.7 (2)
C6—C7—C8—C11	−177.79 (19)	C4—C5—N1—C2	37.7 (2)

### Hydrogen-bond geometry (Å, °)

**Table:**

*D*—H···*A*	*D*—H	H···*A*	*D*···*A*	*D*—H···*A*
O1—H1···I1^i^	0.82	2.80	3.6187 (18)	173.
O2—H2···I1^ii^	0.82	2.67	3.4798 (16)	172.

Symmetry codes: (i) *x*+1, *y*, *z*+1; (ii) −*x*+1, *y*−1/2, −*z*+1.
